# Behaviour of adipose-derived canine mesenchymal stem cells after superparamagnetic iron oxide nanoparticles labelling for magnetic resonance imaging

**DOI:** 10.1186/s12917-017-0980-0

**Published:** 2017-02-24

**Authors:** Malgorzata Anna Kolecka, Stefan Arnhold, Martin Schmidt, Christine Reich, Martin Kramer, Klaus Failing, Kerstin von Pückler

**Affiliations:** 1 Department of Veterinary Clinical Sciences, Clinic for Small Animal–Surgery, Faculty of Veterinary Medicine, Justus–Liebig–University Giessen, Giessen, Germany; 2 Institute of Veterinary Anatomy, Histology and Embryology, Faculty of Veterinary Medicine, Justus–Liebig–University Giessen, Giessen, Germany; 3 Institute of Biomathematics, Faculty of Veterinary Medicine, Justus–Liebig–University Giessen, Giessen, Germany

**Keywords:** Canine adipose-derived mesenchymal stem cells, Superparamagnetic iron oxide particles, Endorem, Magnetic resonance

## Abstract

**Background:**

Therapy with mesenchymal stem cells (MSCs) has been reported to provide beneficial effects in the treatment of neurological and orthopaedic disorders in dogs. The exact mechanism of action is poorly understood. Magnetic resonance imaging (MRI) gives the opportunity to observe MSCs after clinical administration. To visualise MSCs with the help of MRI, labelling with an MRI contrast agent is necessary. However, it must be clarified whether there is any negative influence on cell function and viability after labelling prior to clinical administration.

**Results:**

For the purpose of the study, seven samples with canine adipose-derived stem cells were incubated with superparamagnetic iron oxide nanoparticles (SPIO: 319.2 μg/mL Fe) for 24 h. The internalisation of the iron particles occurred via endocytosis. SPIO particles were localized as free clusters in the cytoplasm or within lysosomes depending on the time of investigation. The efficiency of the labelling was investigated using Prussian blue staining and MACS assay. After 3 weeks the percentage of SPIO labelled canine stem cells decreased. Phalloidin staining showed no negative effect on the cytoskeleton. Labelled cells underwent osteogenic and adipogenic differentiation. Chondrogenic differentiation occurred to a lesser extent compared with a control sample. MTT-Test and wound healing assay showed no influence of labelling on the proliferation. The duration of SPIO labelling was assessed using a 1 Tesla clinical MRI scanner and T2 weighted turbo spin echo and T2 weighted gradient echo MRI sequences 1, 2 and 3 weeks after labelling. The hypointensity caused by SPIO lasted for 3 weeks in both sequences.

**Conclusions:**

An Endorem labelling concentration of 319.2 μg/mL Fe (448 μg/mL SPIO) had no adverse effects on the viability of canine ASCs. Therefore, this contrast agent could be used as a model for iron oxide labelling agents. However, the tracking ability in vivo has to be evaluated in further studies.

## Background

The use of stem cells is becoming increasingly important in veterinary medicine. Mesenchymal stem cells (MSCs) have been shown to improve tissue repair in oral ulcers [1, 2] and bone defects [3–6], as well as in dogs with osteoarthritis of the coxofemoral and elbow joint [7–10]. MSCs have also been used in canine central nervous system to treat spinal cord injury [11–14] and ischemic brain infarction [15].

There is still little information about the exact mechanism of action of MSCs. The behaviour of the MSCs during the stem cell therapy can be examined non-invasively by magnetic resonance imaging (MRI). However, labelling of the stem cells is required in order to distinguish administered cells from the host tissue. A couple of intracellular strategies have been suggested to label MSCs [16–19]. One of them is based on the use of superparamagnetic iron oxide particles (SPIO). The advantage of SPIO particles is that they are taken up via endocytosis as well as by nonphagocytic cells and there is no need for a transfection agent [18, 20, 21]. A commercially available MRI contrast agent that contains a dextran coated SPIO formulation—ferrumoxides—is known under the name Endorem (Guerbet). Endorem affects the T2 relaxation time by inducing a strong field inhomogeneity, leading to a signal decrease as a result of the susceptibility changes in the tissues containing Endorem.

However, it is still unclear whether Endorem labelling has a negative influence on canine MSCs’ viability, proliferation, cytoskeleton and differentiation potential. Another question concerns the duration of the labelling and the amount of contrast agent necessary to preserve detectability of the MSCs via MRI.

This study was designed to prospectively investigate the growth behaviour and MRI signal properties of adipose-derived canine stem cells (ASCs) after labelling with the MRI contrast agent Endorem using 1 Tesla MRI in vitro. The use of 1 Tesla MRI to detect Endorem labelled cells could enable routine examination after stem cell therapy in veterinary clinical practice to verify correct implantation and further distribution of the MSCs.

## Methods

### Isolation of canine mesenchymal stem cells

MSCs were isolated as previously reported [22] from intraabdominal or subcutaneous adipose tissue that was harvested from seven dogs during routine surgical procedures. Fat was removed in order to improve the intraoperative visibility of other organs and was supposed to be disposed. All dogs were free of systemic diseases.

### Labelling of adipose derived mesenchymal stem cells

To assess the optimal labelling concentration of Endorem, three different concentration were tested (10 μL (SPIO = 158 μg/mL; Fe = 112 μg/mL), 28.35 μl (SPIO = 448 μg/mL; Fe = 319.2 μg/mL) and 40 μL (SPIO = 632.4 μg/mL; Fe = 448 μg/mL). These concentrations were chosen according to the results of the doctoral thesis of Kruttwig (2009). An Endorem concentration of 28.35 μL in 1 mL of medium labelled most of the ASCs without comprising their spindle morphology. After labelling with the respective concentrations the live cell imaging observations remained unchanged. For this reason an Endorem concentration of 28.35 μL was used in the present study. ASCs (150 000 cells) were incubated with Endorem at a concentration of 28.35 μl/mL (SPIO = 448 μg/mL; Fe = 319.2 μg/mL) for 24 h.

### Prussian blue staining (PB)

PB staining was performed on all seven samples 3 days, 1, 2 and 3 weeks after Endorem labelling to evaluate if SPIO were incorporated into the cells. Number of blue iron inclusions within the cells at all time intervals was assessed to set a reference value. After incubation the cells were fixed with 4% paraformaldehyde (PF). The PB solution was prepared by mixing 3 mL of 2% potassium ferrocyanide (II) (Merck) with the same amount of 1% hydrochloric acid (Merck). The stain was then put on the cells at room temperature for 20 min. Nuclear fast red-aluminum sulphate staining was performed to colour the nuclei. All of the stained samples were then evaluated under a microscope (Axiophot, Zeiss) at 12.5x magnification.

### Magnetic activated cell sorting (MACS)

MACS cell separation was undertaken to assess the efficiency of the labelling. A MiniMACS separator (Miltenyi Biotec) was used to sort the labelled from unlabelled cell population 1, 2 and 3 weeks after labelling using MS Column guidance (Miltenyi Biotec). After centrifugation both fractions of cells were counted using a Neubauer cell chamber (Marienfeld) to evaluate the percentage of each fraction.

### Phalloidine staining (PS)

PS was performed with all samples 1 week after labelling. The distribution and integrity of the actin filaments of labelled cells was compared to that of unlabelled cells. Seven days after Endorem labelling, the labelled and unlabelled cells were fixed with 4% PF and permeabilized with 1% Triton (Serva). The cytoskeleton was stained with phalloidine (Sigma-Aldrich) and the nuclei contra-stained with Hoechst-33342 (Invitrogen). Fluorescence micrographs were taken with an Axio Observer 2.1 (Zeiss) and a Leica Camera under 100- and 400× magnification.

### Transmission electron microscopy (TEM)

TEM was performed on four samples to assess the intracellular location of Endorem particles. Unlabelled and labelled ASCs 1 and 3 (two samples) or 2 and 3 weeks (two samples) after Endorem labelling were investigated. After incubation in a Chamber Slide System (Thermo Fischer) unlabelled and labelled ASCs were fixed with Yellow-Fix (2% glutaraldehyde (Agar Scientific), 2% PF, and 0.02% picric acid (Fluka Analytical)), then postfixed with 1% osmium solution (Carl Roth). The samples were dehydrated with ethanol (Merck) and embedded in Epon (Serva). Ultrathin sections (70–90 nm) were stained with lead citrate and uranyl acetate (Leica microsystems), collected on copper grids and examined under a TEM 109 (Zeiss). Pictures were taken using a digital camera (Leica Camera DFC320, Leica microsystems).

### Investigation of the influence of Endorem labelling on the multipotency of adipose derived stem cells

#### Adipogenic differentiation

For the evaluation of adipogenic differentiation, samples from two dogs were investigated. ASCs were divided into three groups: labelled, unlabelled and negative control cells. Both labelled and unlabelled cells were incubated in adipogenic medium (DMEM low glucose, Gibco life technologies), 10% FBS (PAA), 1% penicillin/streptomycin (AppliChem), 0.1 μM dexamethasone (Sigma Aldrich), 5 μg/mL ITS (Sigma Aldrich), 0.2 mM indomethacin (Sigma Aldrich) and 0.5 mM IBMX (Sigma Aldrich) for 2 weeks, while negative control cells were incubated in the standard medium. After this time the cell population was fixed in 4% PF and red oil O staining was performed. The nuclei were stained with hematoxyline (Merck) for 10 s. The glass slides with the stained cells were embedded in Kaiser’s glycerol gelatine (Merck) and examined by light microscopy (Leica camera 090135006; Leica Microsystems).

#### Osteogenic differentiation

For the assessment of osteogenic differentiation, two samples were investigated. ASCs were divided as with the adipogenic differentiation into three groups. The labelled and unlabelled cell fractions were incubated in an osteogenic medium (DMEM low glucose, 10% FBS, 1% penicillin/streptomycin, 0.1 μM dexamethasone, 10 mM ß-glycerol phosphate (Sigma Aldrich), 0.06 mM ascorbic acid (Sigma Aldrich)) for 3 weeks, while the cells of the negative control were incubated in the standard medium. After fixation with 4% PF, von Kossa staining was performed. Cells were incubated in a 5% silver nitrate solution (Merck) for 30 min and then with 5% natrium carbonate formaldehyde solution (Merck) for 5 min. After an incubation with Farmer’s reducer for 30 s, the nuclei were stained with nuclear fast red-aluminium sulphate. Embedding was then performed corresponding to adipogenic differentiation.

#### Chondrogenic differentiation

For the evaluation of chondrogenic differentiation potential, two samples were investigated. ASCs were divided into three groups: labelled, unlabelled and negative control cells. Both the labelled and unlabelled cells were incubated in chondrogenic medium ((DMEM low glucose, 1% penicillin/streptomycin, 0.1 μM dexamethasone, 10 μg/mL ITS (Sigma Aldrich), 0.9 mM natrium pyruvate (Sigma Aldrich), 0.17 mM ascorbic acid, 0.35 mM proline (Sigma Aldrich), TGF – ß (Sigma Aldrich)) for 4 weeks in 15 mL falcon tubes, while negative control cells were incubated in standard medium. After 4 weeks the entire cell population was fixed in 4% PF, embedded in paraffin and cut and stained with Alcian blue (Merck).

### 3-(4,5-Dimethylthiazol-2-yl)-2,5-diphenyltetrazoliumbromid (MTT) test

MTT test was performed on two samples to assess the influence of the Endorem labelling on the proliferation of the labelled cells. Stem cells were seeded at a density of 28 500 cells/ well in two 24 well plates. Half of the cells were labelled with Endorem, then incubated for 24 h, second plate 48 h, respectively. After this time 0.5 mg/mL MTT solution (Sigma Aldrich) was added to one of the plate for 4 h, then 200 μL DMSO (Applichem) for 10 min. The absorption of the labelled and unlabelled cells was measured with Tecan sunrise (Tecan) 24 and 48 h after labelling.

### Wound healing and migration

Migration and Proliferation of the labelled and unlabelled cells of 5 samples were further investigated using 2 well Culture inserts system (IBIDI) according to the guidelines. After 24 h incubation the culture insert was removed. Life cell imaging (Zeiss) was used to evaluate the “wound healing process” over 24 h. The evaluation of the cell uncovered area was assessed using Image J Software (National Institute of Health, USA) every 3 h over 24 h period.

### Investigation of labelling efficiency via MRI

**Fig. 1:**
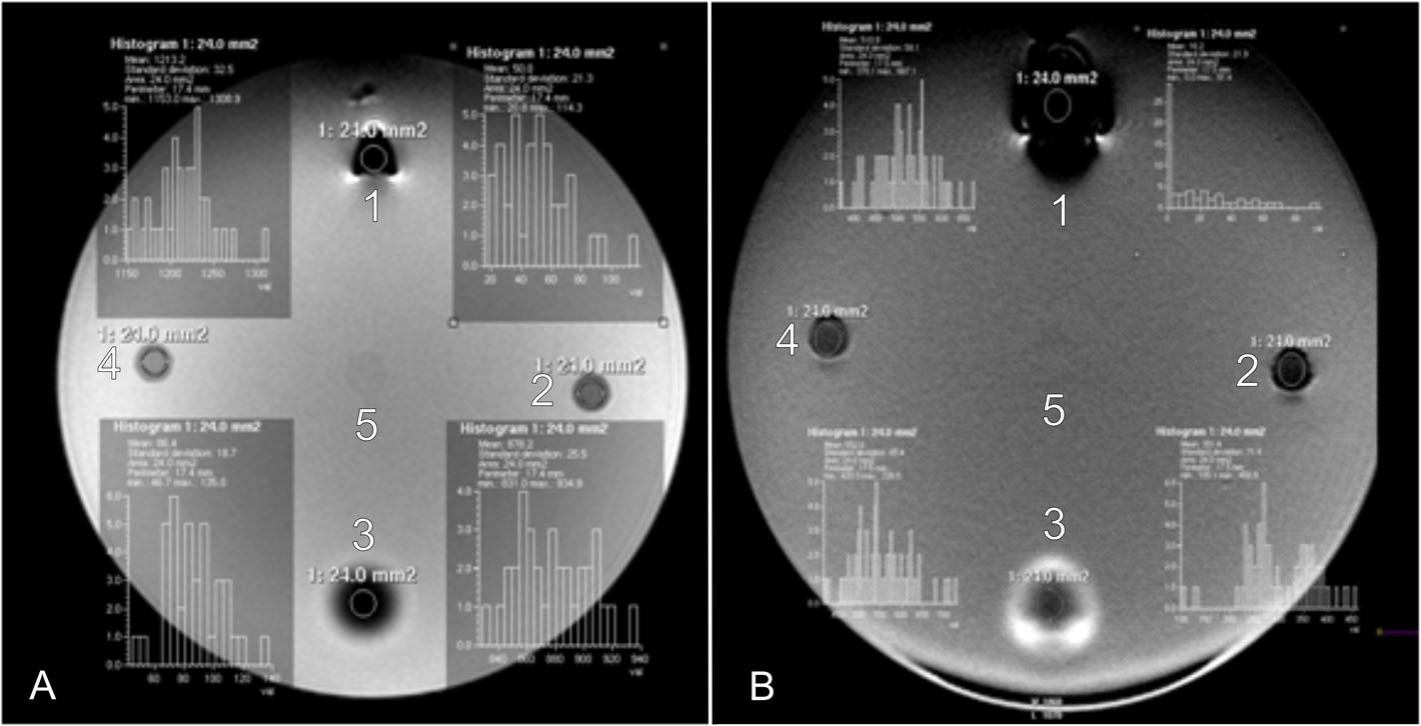
Agar phantom scanned in the T2 TSE (**a**) and T2 FFE (**b**) with histograms showing measurement of the signal intensity of regions of interest (ROI) containing: (1) SPIO (pure Endorem); (2, 4) SPIO labelled ASCs; (3) Gadolinium MRI contrast agent (Dotarem); (5) negative control containing agarose gel only

For the investigation of the labelling efficiency a 1 Tesla clinical MRI scanner (Gyroscan Intera, Philips Medical Systems) was used. Endorem labelled cells from all seven samples were placed into 0.5 ml 0.6% agar solution and then in an agar phantom 1, 2 and 3 weeks after labelling. The mean cell count was 1 800 857, 2 125 000, 2 538 928 for each week respectively. Additionally, a negative control containingagar gel solely, a positive control with Endorem (concentration 28.35 μL/mL) and a control with the positive contrast agent Dotarem (Guerbert) were investigated. A horizontally oriented gradient echo (T2w FFE) (field of view 140 mm, repetition time 200 ms, time of echo 21 ms, 17 slices, thickness 1.5 mm /-0.9 mm, flip angle 30°) and a turbo spin echo sequence (T2w TSE) (field of view 200 mm, repetition time 6000 ms, time of echo 71 ms, 14 slices, thickness 1.6 mm /-1 mm) were performed. The voxel size and number of voxels per sample can be found in the Table 1. For the relative estimation of the signal intensity a dedicated software (View Forum R6.3V1L7 SP1 2010, Philips Medical System) was used. The signal intensity was measured for every sample (agar with labelled cells, negative control, agar with Endorem). For this purpose a region of interest (ROI) of 24 mm^2^ was placed in every sample. A graph illustrates the signal intensity and its frequency within every sample and also maximal, minimal and mean values including the standard deviation (Fig. 1).

**Table 1:** Acquired, reconstructed voxel size (mm) and number of voxels per sample (24 mm^2^)

Sequence	Acquired Voxels (mm)	Reconstructed Voxels (mm)	Voxels per sample (24 mm2)
T2FFE	0.55 x 0.55 x 1.5	0.55 x 0.55 x 1.5	43.64
T2wTSE	0.69 x 0.82 x 1.6	0.69 x 0.69 x 1.6	34.78

### Statistical analysis

One-way analysis of variance with repeated measurements was performed to assess the decrease in the labelled cells in the MACS test over 3 weeks. To determine reference values of the labelled cells 1, 2 and 3 weeks after labelling in the MACS test and the value of the number of Endorem particles in a single cell in the Prussian blue staining, a 95% confidence interval was calculated.

Influence of Endorem labelling in the migration und wound healing assay was performed using *t*-test. Further, the influence of the cell line on the wound healing process was assessed using Pearson correlation test.

The development of the signal intensity of the labelled cells in MRI was assessed with 2-way analysis of variance (ANOVA) with repeated measurements with respect to the MRI sequence and the point in time.

Furthermore, correlation analysis with the Pearson’s correlation coefficient was performed between the signal intensities of both MRI sequences and the MACS test for 1, 2 and 3 weeks after labelling.

## Results

### Isolation of ASCs and efficiency of Endorem labelling in the Prussian blue staining and MACS assay

**Fig. 2:**
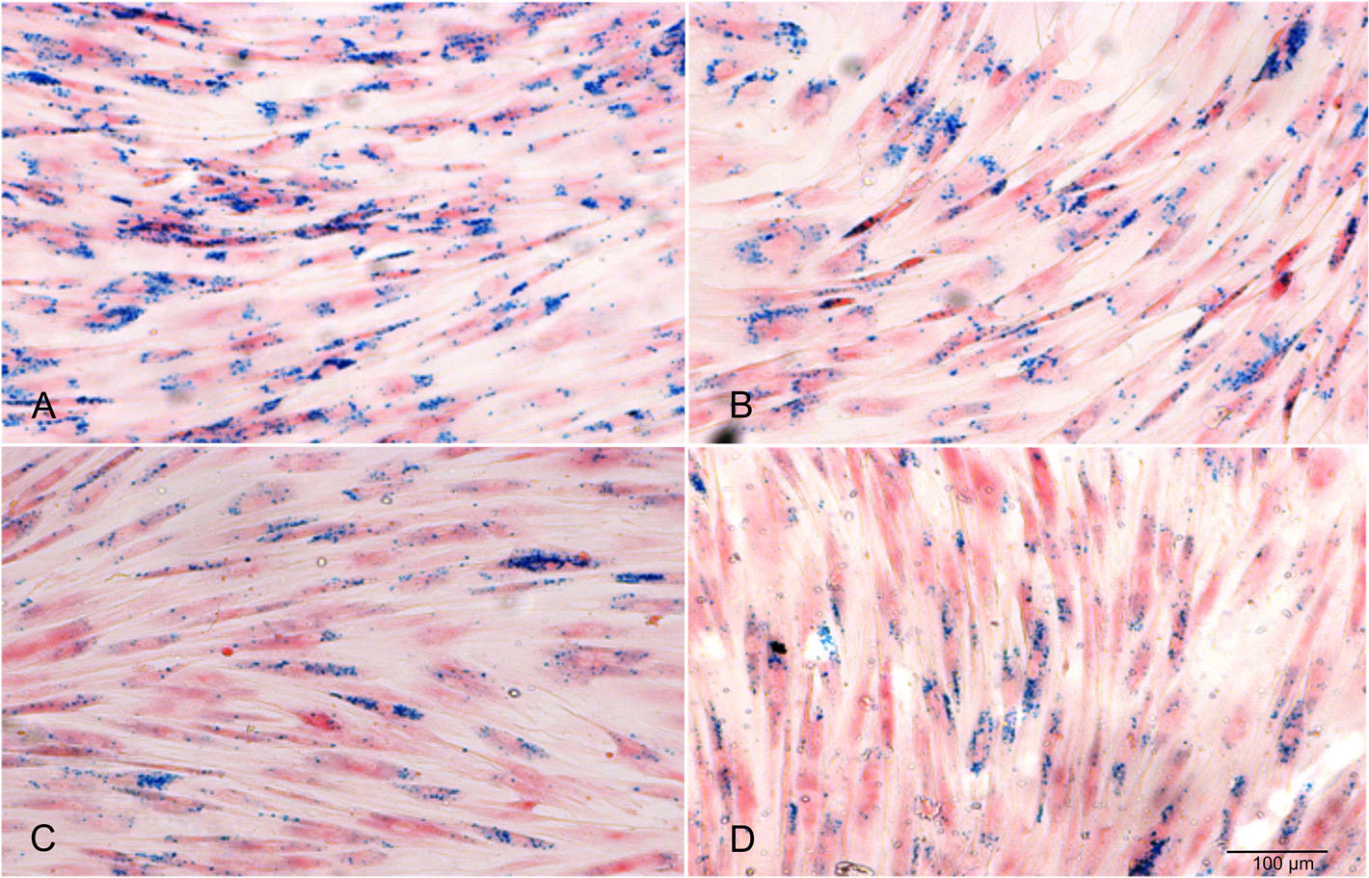
Prussian Blue staining of SPIO-labelled canine ASCs shows blue iron particles within the cells after SPIO labelling. **a** Three days. **b** One week. **c** Two weeks. **d** Three weeks

ASCs could be easily isolated from canine adipose tissue. After 24 h incubation plastic adherent cells with fibroblastic morphology could be observed. Three days after labelling with PB there were numerous blue iron inclusions visible within the cells (Fig. 2a). An Endorem concentration of 448 μg/mL SPIO was sufficient to visually label all cells within 24 h. Iron particles were still detectable in the cells 3 weeks after labelling with Endorem (Fig. 2d). The iron particles were not equally subdivided to the daughter cells during cell division. It was not possible to set a reference value of iron particles within a single cell at time intervals of three, seven, fourteen and twenty one days with a 95% confidence interval calculation.

In the MACS assay, one week after labelling the labelled cells accounted for: 92.36%, 91.28%, 92.78%, 87.5%, 84.04%, 46.3% and 25.4% of all cells in samples one – seven, respectively; 2 weeks after labelling: 70.71%, 70.2%, 71.64%, 62.27%, 61.97%, 32.4%, 18.3%; and 3 weeks after labelling: 68.79%, 78.53%, 59%, 73.4%, 20.33%, 27.7%, 11.1%. It was not possible to determine reference values of the percentage of labelled cells for the time intervals of 1, 2 and 3 weeks.

### Influence of the Endorem labelling on the cytoskeleton

**Fig. 3:**
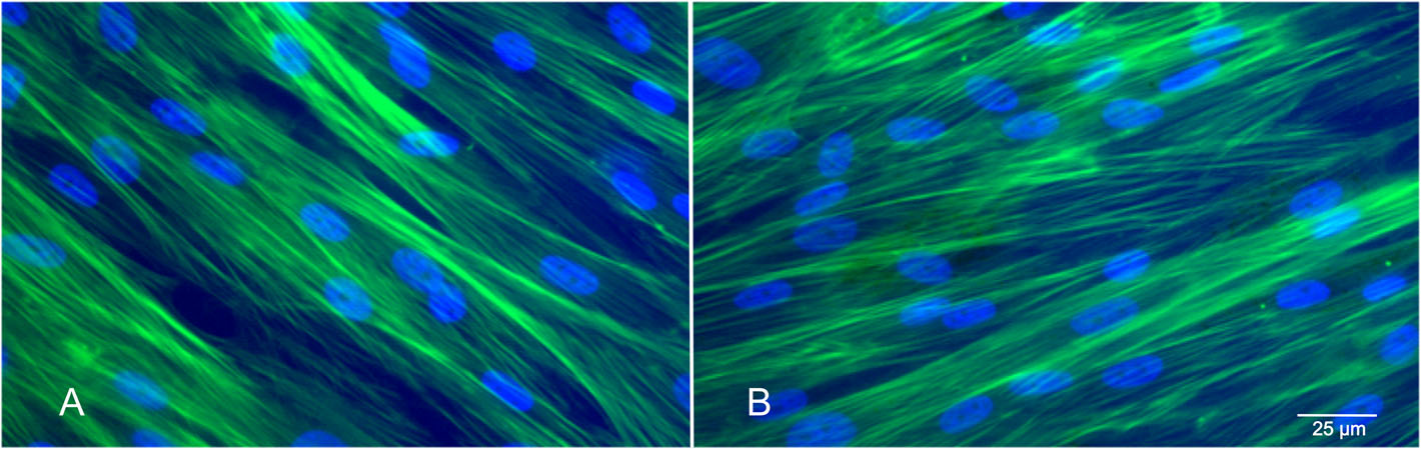
Phalloidin staining shows no alteration of the green actin filaments of the cytoskeleton after labelling (**b**) comparing with unlabelled ASCs (**a**)

A comparison of the integrity and distribution of actin filaments in the labelled and unlabelled cells under 100 and 400 times magnification revealed no alterations (Fig. 3a, b).

### Transmission electron microscopy (TEM) examination: distribution of the Endorem particles within the cell

**Fig. 4:**
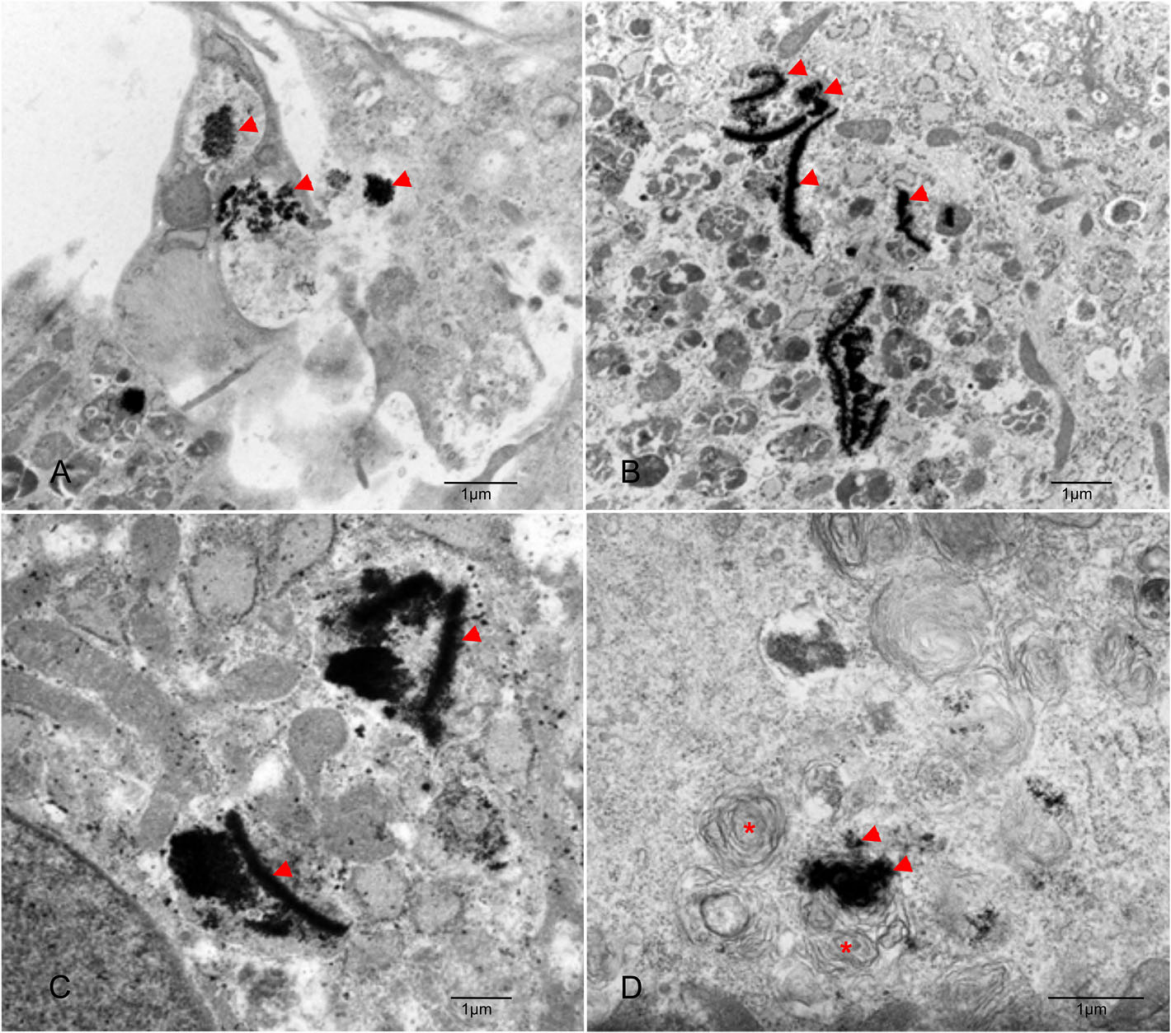
Transmission electron microscopy (TEM) of SPIO labelled ASCs showing black iron inclusions (*red arrows* ) in the cytoplasm. **a** Capture of the moment of endocytosis of SPIO into a cell and formation of a membrane vesicle. **b** Cluster of SPIO particles within the cytoplasm one week after labelling. **c** Cluster of SPIO particles within the cytoplasm two weeks after SPIO labelling. (**d**) SPIO cluster within lysosomes (*asterisk* ) three weeks after labelling

Under TEM, SPIO particles were seen within the cells and also within vesicles outside the cells (Fig. 4 a-d). The moment of endocytosis of SPIO into the cell and formation of a membrane vesicle could also be captured (Fig. 4 a). One and two weeks after labelling the iron clusters were mainly visible within the cytoplasm without compartmentalisation (Fig. 4 b, c). In week 3 the Endorem particles were basically detected in lysosomes (Fig. 4d).

### Influence of the Endorem labelling on multipotency, proliferation and viability

**Fig. 5:**
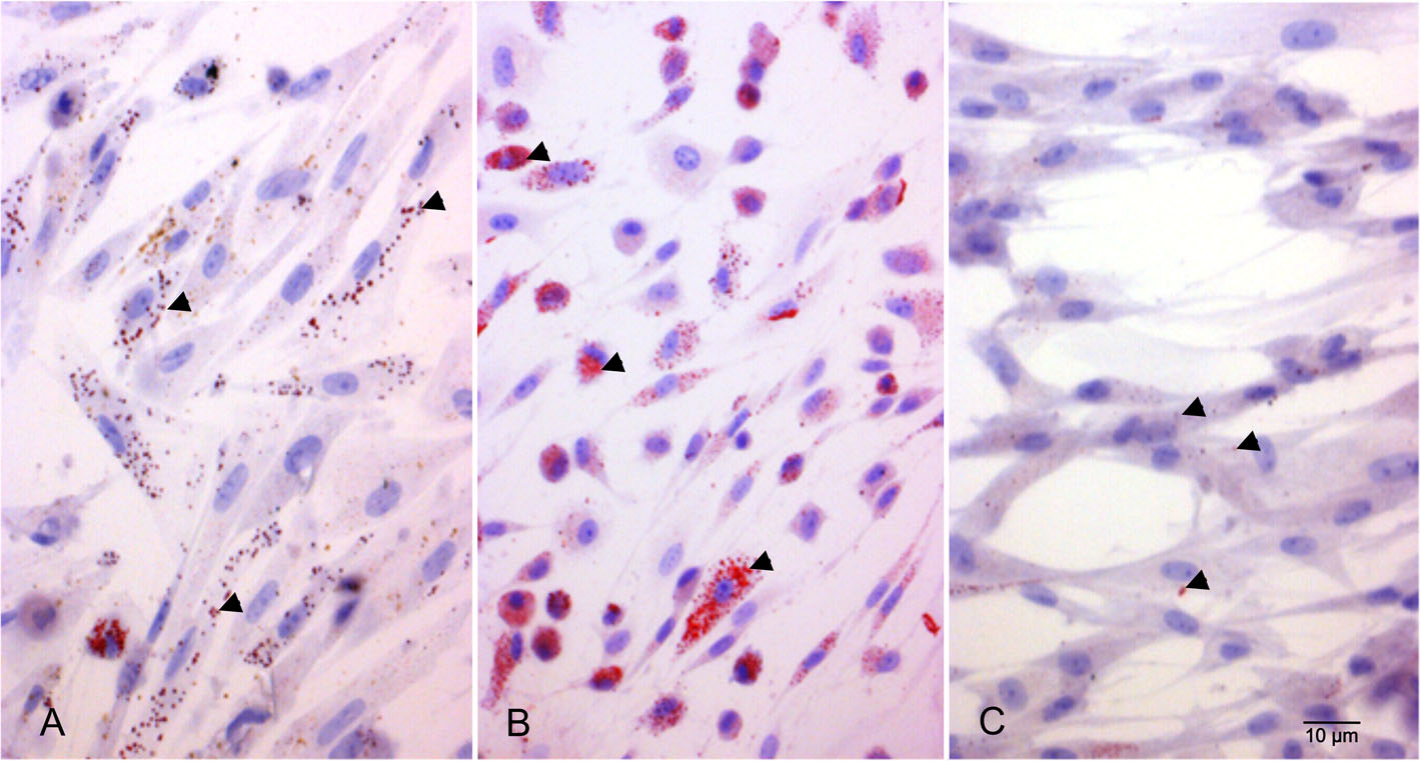
Red Oil O staining shows red lipid vacuoles (*arrows* ) in (**a**) Unlabelled ASCs cultured in adipogenic medium. **b** SPIO-labelled ASCs cultured in adipogenic medium. **c** Control with unlabelled ASCs incubated in standard medium

**Fig. 6:**
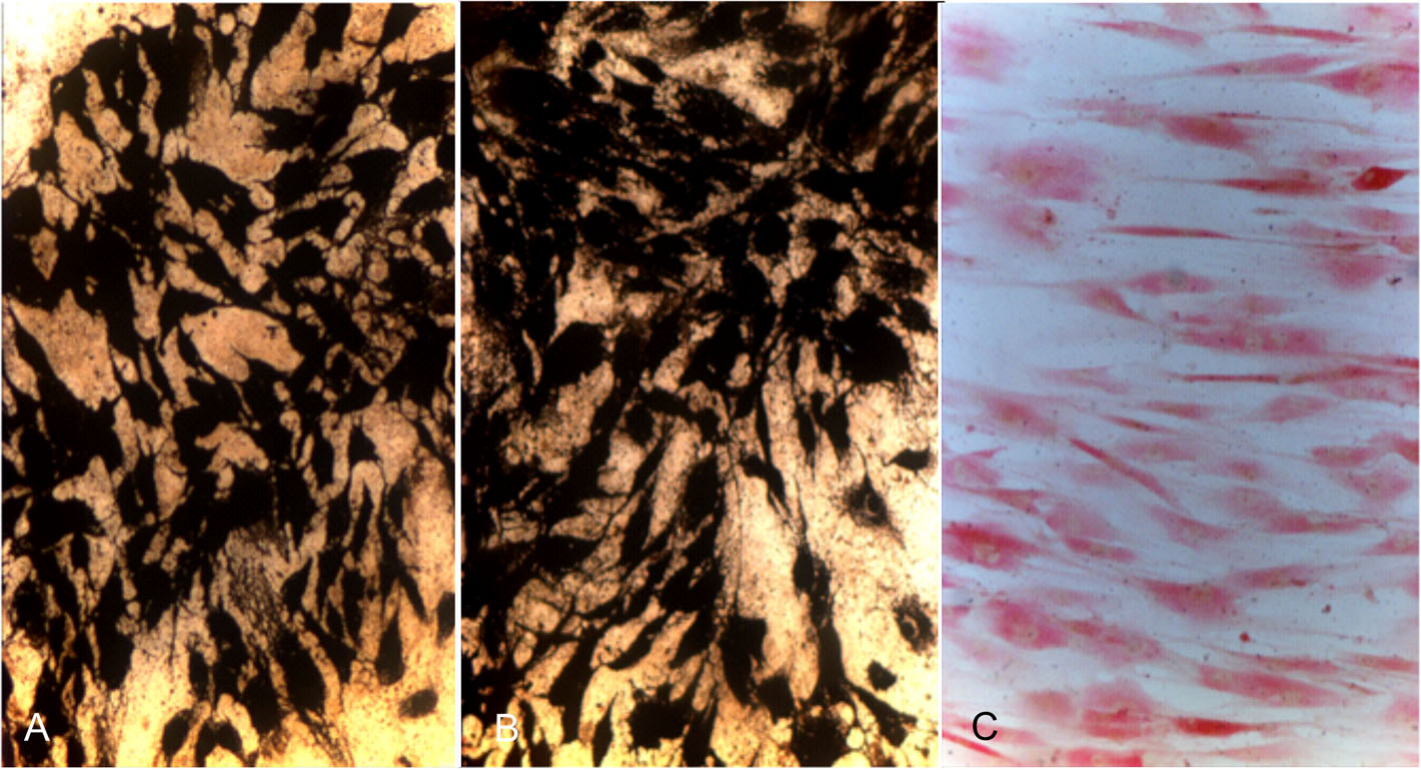
Von Kossa staining shows black stained mineralised extracellular matrix. Note also the black staining of the cell bodies. **a** Unlabelled ASCs incubated in osteogenic medium. **b** SPIO-labelled ASCs incubated in osteogenic medium. **c** Control with unlabelled ASCs incubated in standard medium

**Fig. 7:**
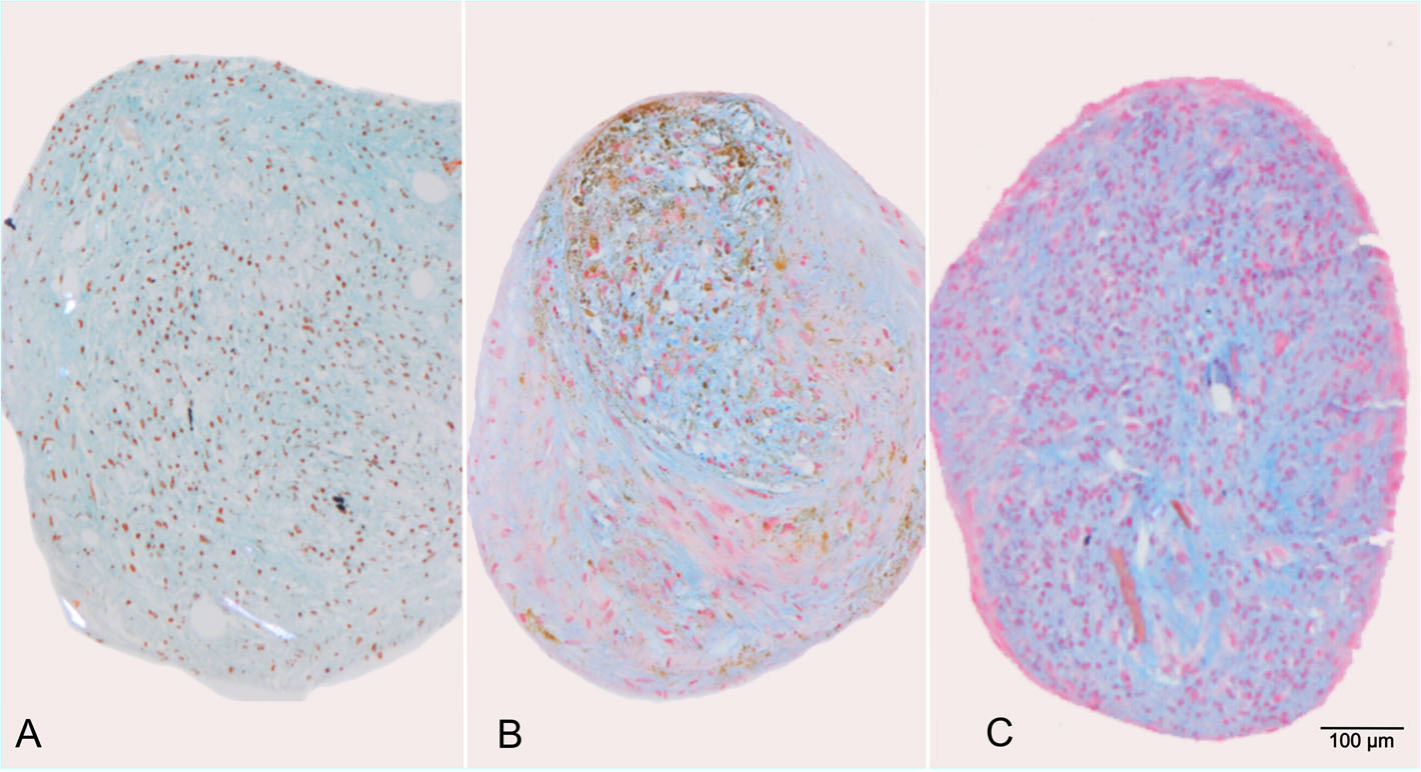
Alcian blue staining shows blue stained proteoglycans of the cartilage matrix. **a** Unlabelled ASCs cultured in chondrogenic medium underwent completed chondrogenesis. **b** SPIO-labelled ASCs cultured in chondrogenic medium show chondrogenesis to a lesser extent than unlabelled cells. **c** Control with unlabelled ASCs incubated in standard medium also show signs of chondrogenesis

Multipotency was assessed on the basis of adipogenesis, chondrogenesis and osteogenesis. Labelled and unlabelled cells incubated with adipogenic medium underwent adipogenic differentiation. Red Oil O stained cells showed multiple red fat vacuoles inside the cells (Fig. 5a, b). Labelled cells also underwent normal osteogenic differentiation. Under von Kossa staining, black stained precipitates in the extracellular matrix could be observed (Fig. 6a, b). The cell bodies also showed a black staining pattern. The control group exhibited no signs of mineralisation (Fig. 6c). In contrast to this, less labelled cells underwent chondrogenic differentiation comparing to the control (Fig. 7b). Interestingly, the negative control also showed signs of chondrogenesis (Fig. 7c).

In MTT test both samples 48 h after labelling showed a higher absorbance (unlabelled cells: sample one: 0.613; 0.480; 0.539; sample two: 0.501; 0.520; 0.486; labelled cells: sample one: 0.786; 0.723; 0.712; sample two: 0.506; 0.396; 0.416) compared to samples 24 h after labelling (unlabelled cells: sample one: 0.079; 0.076; 0.071; sample two: 0.087; 0.080; 0.091; labelled cells: sample one: 0.096; 0.096; 0.071; sample two: 0.068; 0.052; 0.065).

Using the wound and healing assay it could be shown that there was no negative influence on cell migration and proliferation after SPIO labelling (*P* = 0.023). Furthermore, it could be demonstrated that there was a donor specific influence on cell migration and proliferation rather than an influence exerted by cell labelling.

### Investigation of labelling efficiency via magnetic resonance imaging

The hypointensity caused by Endorem lasted for 3 weeks in both sequences. The signal intensity for both sequences changed within the 3 week time period. These changes were significant (*P* = 0.003). A statistical correlation between the MACS test for 1, 2 and 3 weeks and the signal intensity in the T2 TSE for 1, 2 and 3 weeks was assessed (week 1: *P* = 0.945; *r* = -0.032; week 2: *P* = 0.242; *r* = -0.510; week 3: *P* = 0.288; *r* = -0.469) and for signal intensity in the T2 FFE (week 1: *P* = 0.722; *r* = -0.166; week 2: *P* = 0.619; *r* = -0.231; week 3: *P* = 0.077; *r* = -0.705).

The values of signal intensity in the T2w TSE and T2w FEE for 1, 2 and 3 weeks were correlated at each point in time (week 1: *P* = 0.037, *r =* 0.784; week 2: *P* = 0.006, *r =* 0.9; week 3: *P* = 0.006, *r =* 0.896) and for whole time period (*P* < 0.001; *r =* 0.879).

It was still possible to detect all of the samples of Endorem labelled stem cells with 1 Tesla MRI in the T2 TSE und T2 FFE sequences 3 weeks after labelling.

## Discussion

The purpose of this study was the development of a labelling strategy for tracking canine ASCs using a clinical 1 Tesla magnetic resonance tomograph. We evaluated the survival, differentiation potential and tracking ability of MSCs after labelling with the contrast agent Endorem.

A number of labelling strategies with different contrast agents have already been used for tracking studies; however, these used MR scanners with a higher magnetic field strength, between 3 and 7 Tesla [19, 23–25]. In most veterinary facilities only low-field MRI is available. We decided to analyse stem cells over a 21 day period of time, assuming that this time frame would be relevant for the distribution of MSCs after clinical application.

Because stem cell therapy has increasingly gained importance for use in veterinary medicine it is essential to develop an easy strategy for cell labelling that has no potential negative influence on cell function. ASCs were used in this study because of their uncomplicated isolation and high number of MSCs in fat tissue. Incubation of canine MSCs with an Endorem concentration of 319.2 μg/mL iron for 24 h resulted in sufficient uptake and labelling of all cells. No transfection agent was used in this study. There have been several studies using different labelling protocols of SPIO with other concentrations and incubation times from 1 till 72 h. Longer incubation times and higher iron concentrations resulted in a negative influence on the labelled cells [26].

The spontaneous uptake of SPIOs from medium containing Endorem was confirmed with PB staining (Fig. 2a-d). Prussian blue staining is a reliable qualitative method to show Endorem particles within the cells. However, iron particles were not equally subdivided to the daughter cells during cell division what caused a substantial variation of the amount of iron particles within a single cell. Therefore, assessment of the reference values using a 95% confidence interval was not possible. Separation of the labelled cells from the unlabelled cells turned out to be a reliable quantitative method. With the aid of the MACS assay it could be shown that the percentage of labelled canine stem cells decreased within three weeks (*P* = 0.0007). Due to variation of the percentage of labelled cells it was not possible to set reference values for MACS test. The last two samples showed considerably lower labelling efficiency. These samples were investigated at the same time. There might be other influential factors as the composition of the medium or incubation protocol. However, this can be considered unlikely as we maintained the same condition during all parts of the study according to the standardized protocol. Another factor might be the variability of the stem cells behavior. As we showed in the migration and wound healing assay the properties of an individual cell line has a more significant influence on the proliferation and migration than labelling. It can be possible that these stem cells did not incorporate SPIO particles to the same extent as other samples or the cells incorporated the SPIO particles but later released them into the medium.

Internalisation of the Endorem particles could also be confirmed using TEM. One and two weeks after labelling the iron clusters were mostly observed distributed within the cytoplasm (Fig. 4b, c). In contrast to that, three weeks after labelling Endorem particles were generally detected in the lysosomes and fewer non-compartment bound iron clusters were seen in the cytoplasm (Fig. 4d). This observation indicates that lysosomes are involved in the dissipation of Endorem and this process starts already two weeks after labelling. Previous studies reported that cluster of iron particles are surrounded by cell membrane structures directly after labelling but further fate of the iron cluster was not investigated [18, 23, 27]. There were no reports about free iron clusters within the cytoplasm. Investigation of the exact localisation of iron particles within the cells is very important due to the fact that their distribution is suspected to have a significant influence on the contrast induced signal void during the MRI examination. Larger iron clusters are responsible for a stronger signal void than the same amount of iron distributed evenly within the cell [17].

Phalloidin staining showed no negative influence of the iron particles on the cytoskeleton (Fig. 3b). This is of great importance considering the essential role of the cytoskeleton in migration capability and cell division. There are reports describing a negative influence of Endorem labelling on the cytoskeleton in rat and human MSCs using a labelling concentration of 600 μg/mL Fe [28].

In contrast to the unaffected osteoegenic and adipogenic differentiation, after Endorem labelling fewer stem cells underwent chondrogenesis compared to a control. Other groups have come to a similar conclusion: a dose dependent inhibition of chondrogenesis in human cells was observed [29, 30]. In contrast, there are also reports about it lacking negative influence on cartilage formation in human cells [31]. We also observed signs of chondrogenesis in the negative control. This phenomenon has already been seen in bovine MSCs. Three-dimensional pellet formation can lead to spontaneous chondrogenesis [32].

It has been shown that human cells were able to proliferate normally after Endorem labelling [31, 33]. Using an MTT test and migration and wound healing assay we could demonstrate that the proliferation of the cells after labelling was not altered [34].

It could be shown in T2 TSE and T2 FFE sequences that the hypointensity caused by Endorem decreased within 3 weeks, but after this time a sufficient signal void could still be seen in the scans. However, the results of the statistical analysis between the percentage of labelled cells in the MACS test for 1, 2 and 3 weeks and the signal intensity in the T2 TSE and T2 FFE sequences for the same points in time showed no statistical correlation. A possible explanation could be that it is not the number of labelled cells but the intracellular distribution of the Endorem particles that is predominantly relevant. With the help of MACS test a differentiation between labelled and unlabelled cells can be made. However, the labelled fraction can also contain cells with a low particles’ number. According to this fact that results in a high variation of the samples, a statistical correlation is not possible. We assumed that Endorem dissipates within canine ASCs within a 3 week period because of cell division and intracellular metabolism. The leakage of iron oxide from the cell to the medium was not investigated in this study but another author reported about this issue [18]. Even if the contrast agent is spontaneously released by the cells there is still enough signal void in T2 FFE and TSE caused by labelled cells after 3 weeks. However, this leakage could represent a limitation for in vivo tracking studies.

The limitation of the current study is the in vitro design. There might be a strong influence of the surrounding structures on the migration and imaging of cells in vivo. However, a few studies that present tracking of SPIO labelled cells in vivo in canine models exist. SPIO labelled canine MSCs administered into renal artery were detectable as hypointense areas in the renal cortex over 8 days at 3 Tesla [35]. Intra-arterial delivered SPIO labelled MSCs in the canine stroke model were seen at least over 4 weeks period at 3 Tesla [36]. Also human SPIO labelled ASCs can migrate to the site of the lesion after radiofrequency ablation and differentiate into cardiomyocyte-like cells [37].

**Fig. 8:**
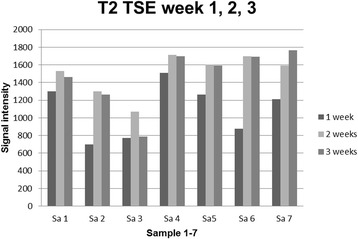
Development of the signal intensity in the T2 TSE sequence for all samples one, two and three weeks after labelling

**Fig. 9:**
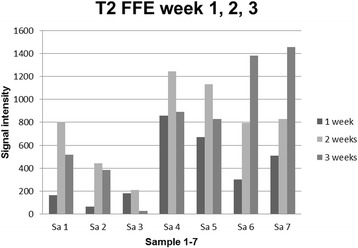
Development of the signal intensity in the T2 FFE sequence for all samples one, two and three weeks after labelling

Because of the decreasing level of Endorem particles in the cells, we assumed that the degree of signal cancellation should decline within a 3 week period and signal increase should be noticed. However, for the 3 week period we observed a decrease in the signal intensity (Figs. 8 and 9). Considering the findings of the TEM investigation after 3 weeks that most Endorem particles were packed in the lysosomes (Fig. 4d) and not distributed within the cell could be the most probable explanation. As already mentioned, this could lead to a stronger signal void. Other studies have also reported that micro air deposits in the agar phantoms can cause artefacts and strengthen the hypointense signal [18]. However, we did not observe any air inclusions in the present study.

We chose T2 TSE and T2 FFE sequences and did not observe relevant differences in in vitro investigation in agar phantoms. In any case, other studies have shown the superiority of the T2 FFE for tracking studies in vitro and in vivo [18, 19, 38]. We decided to use also T2 TSE as this is a standard sequence superior to T2 FFE concerning the clinical use due to the clear depiction of the anatomy with high contrast between different soft tissues. The aim was to investigate whether the influence of the iron oxides is strong enough to influence also the T2 spin echo signal. The advantage of an investigation using the T2 TSE sequence would be the picture of the anatomy and assessment of the iron distribution at the same time. However, T2 FFE sequences are more sensitive regarding the paramagnetic properties of the iron oxide nanoparticles. This is why the T2 FFE sequence should be the method of choice to detect Endorem labelled stem cells.

## Conclusion

An Endorem labelling concentration of 319.2 μg/mL Fe (448 μg/mL SPIO) had no adverse effects on the viability of canine ASCs. After a 3 week period of culture Endorem labelled ASCs were detectable with a 1 Tesla magnetic resonance tomograph in T2w TSE and T2wFFE sequences. Therefore, this contrast agent could be used as a model for iron oxide labelling agents. However, the tracking ability in vivo has to be evaluated in further studies. A potential influence on chondrogenesis should be further investigated.
